# Occupational Histoplasmosis: Epidemiology and Prevention Measures

**DOI:** 10.3390/jof7070510

**Published:** 2021-06-26

**Authors:** Marie A. de Perio, Kaitlin Benedict, Samantha L. Williams, Christine Niemeier-Walsh, Brett J. Green, Christopher Coffey, Michelangelo Di Giuseppe, Mitsuru Toda, Ju-Hyeong Park, Rachel L. Bailey, Randall J. Nett

**Affiliations:** 1 Office of the Director, National Institute for Occupational Safety and Health, Centers for Disease Control and Prevention, Cincinnati, OH 45226, USA; 2 Division of Foodborne, Waterborne, and Environmental Diseases, National Center for Emerging and Zoonotic Infectious Diseases, Centers for Disease Control and Prevention, Atlanta, GA 30333, USA; kbenedict@cdc.gov (K.B.); swilliams21@cdc.gov (S.L.W.); mtoda@cdc.gov (M.T.); 3 Division of Field Studies and Engineering, National Institute for Occupational Safety and Health, Centers for Disease Control and Prevention, Cincinnati, OH 45226, USA; cuebel@cdc.gov; 4 Health Effects Laboratory Division, National Institute for Occupational Safety and Health, Centers for Disease Control and Prevention, Morgantown, WV 26505, USA; bgreen1@cdc.gov; 5 National Personal Protective Technology Laboratory, National Institute for Occupational Safety and Health, Centers for Disease Control and Prevention, Pittsburgh, PA 15236, USA; ccoffey@cdc.gov (C.C.); mdigiuseppe@cdc.gov (M.D.G.); 6 Respiratory Health Division, National Institute for Occupational Safety and Health, Centers for Disease Control and Prevention, Morgantown, WV 26505, USA; jpark2@cdc.gov (J.-H.P.); rlbailey@cdc.gov (R.L.B.); rnett@cdc.gov (R.J.N.)

**Keywords:** Histoplasmosis, *Histoplasma*, occupational, workers, fungal

## Abstract

In areas where *Histoplasma* is endemic in the environment, occupations involving activities exposing workers to soil that contains bird or bat droppings may pose a risk for histoplasmosis. Occupational exposures are frequently implicated in histoplasmosis outbreaks. In this paper, we review the literature on occupationally acquired histoplasmosis. We describe the epidemiology, occupational risk factors, and prevention measures according to the hierarchy of controls.

## 1. Introduction

Histoplasmosis is an infection caused by inhalation of conidia of the fungus *Histoplasma*. First described by Samuel Darling in 1906 [1], two varieties of *Histoplasma capsulatum* are recognized as the etiological agents of histoplasmosis in humans and include *H. capsulatum* var. *capsulatum* and *H. capsulatum* var. *duboisii* [2,3]. *H. capsulatum* belongs to the family, Ajellomycetaceae (order Onygenales), that include a unique group of pathogenic fungi that produce a thermally dimorphic yeast phase [4]. In the environment, *H. capsulatum* grows as a saprobic filamentous form and is composed of septate hyphae [5]. *H. capsulatum* produces two types of asexual conidia [6]; tuberculate macroconidia (8–15 µm) and microconidia (2–4 µm) [2,7]. Although there are reported differences between *H. capsulatum* strains, microconidia are the main conidia produced on the hyphae [7]. Abiotic or biotic disturbance to *H. capsulatum* colonies during occupational activities may result in the aerosolization and potential inhalation of microconidia by the worker [8]. In vivo deposition of microconidia and introduction to physiological temperature profiles results in the conversion of *H. capsulatum* microconidia to the pathogenic polar budding yeast phase (2–4 µm) [5]. The dimorphic switch of *H. capsulatum* helps to subvert the host immune defenses and may result in systemic infections due to intra-phagosomal adaptations that allow the yeast to persist and proliferate within intracellular environments, such as macrophage phagolysosomes [5,9].

*H. capsulatum* var. *capsulatum* has a nearly worldwide distribution but appears to be most common in the central and eastern United States, particularly around the Ohio and Mississippi River Valleys [10]. People in these areas are at risk for inhaling *Histoplasma* from the environment, especially those who have occupations or participate in activities exposing them to soil that contains bird or bat droppings [11]. Occupational exposures are frequently implicated in histoplasmosis outbreaks [11].

In this paper, we review the literature on occupationally acquired histoplasmosis. We describe the epidemiology in the United States, occupational risk factors, and prevention measures according to the hierarchy of controls.

## 2. General Epidemiology

Anyone can acquire histoplasmosis in areas where *Histoplasma* is present in the environment. An estimated 60% to 90% of people who live in areas surrounding the Ohio and Mississippi River valleys have been exposed to *Histoplasma* at least once during their lifetime [12]. Most people who are infected never experience symptoms. Symptomatic histoplasmosis, although it is likely vastly under-recognized, appears to account for only a small fraction of total infections, and depends on both the host’s immune status and the extent of the exposure. Symptomatic infections are associated with nonspecific symptoms such as cough, fever, or shortness of breath, typically following a 3–17-day incubation period [13]. Infection is often clinically indistinguishable from other respiratory illnesses, such as community-acquired pneumonia, and nonspecific symptoms can lead to diagnostic delays or ineffective treatment [14,15].

In some cases, particularly among immunocompromised people, histoplasmosis can result in severe illness, including pulmonary infection or disseminated disease [15,16]. People with HIV/AIDS, organ transplant recipients, and those taking immunosuppressive agents such as corticosteroids are at greater risk for developing severe histoplasmosis [16,17,18]. Severity can also be associated with exposure intensity [15].

Understanding the geographic distribution of histoplasmosis is helpful in targeting prevention and control measures. Large-scale skin-testing studies performed in the 1940s and 50s not only provided the basis for the organism’s approximate geographic distribution, but also confirmed widespread asymptomatic infection with *Histoplasma* based on the high prevalence of positive reactions to *Histoplasma* antigen in certain areas [12,19,20]. Based on public health surveillance, environmental data, and outbreak investigations, the geographic distribution of histoplasmosis is likely wider than currently recognized [10,21]. Similarly, estimates of case counts and incidence are likely subject to underreporting and misdiagnosis [22].

## 3. *Histoplasma* in the Environment

*Histoplasma* has a nearly worldwide distribution but is most common in North America and Central America [10]. In the United States, *Histoplasma* mainly lives in the central and eastern states, particularly areas around the Ohio and Mississippi River Valleys. However, sporadic histoplasmosis cases in humans and animals in places like Alaska, California, and Florida indicate that *Histoplasma* can also survive in other areas given suitable environmental conditions [21]. For example, *Histoplasma* grows especially well in soil or other environmental material containing large amounts of bird or bat droppings, although this is not a requirement for its presence in the environment. *Histoplasma* has been detected in some organic fertilizers in Latin America, but more studies are needed to understand whether the fungus can survive commercial fertilizer manufacturing processes [23,24].

In the environment, *Histoplasma* is undetectable to the naked eye. Laboratory testing to detect *Histoplasma* in environmental samples can be challenging and is not routinely recommended because it is unlikely to be useful without a strong epidemiologic hypothesis to guide the sampling strategy. In addition, a positive result may not change public health recommendation to prevent future histoplasmosis cases in a setting that poses a clear risk, and a negative result may not necessarily mean that *Histoplasma* is not present or was not present in the past. However, focused PCR or culture testing can sometimes help confirm a suspected environmental source during a histoplasmosis outbreak.

## 4. Activities, Settings, and Jobs Associated With Histoplasmosis

In areas where *Histoplasma* is endemic in the environment, occupations involving activities exposing workers to soil that contains bird or bat droppings may pose a risk for histoplasmosis. In particular, this includes people who work in construction and extraction occupations and in agriculture, forestry, and hunting industries [11,15]. People who are exposed to bird or bat droppings and work in these and other occupations or industries are also likely at higher risk for contracting histoplasmosis.

Most of the information about the types of exposures associated with histoplasmosis has been derived from outbreak investigations. In the United States, more than 100 histoplasmosis outbreaks have been described in published literature since 1938, comprising nearly 3000 cases [11]. This almost certainly underestimates the true number of histoplasmosis outbreaks that occurred because many go undetected, are not reported to public health authorities, are not investigated, or the investigation findings are not published. In addition, histoplasmosis outbreaks comprise only a small proportion (~5%) of all reported histoplasmosis cases [15,25]. Nevertheless, investigations of histoplasmosis outbreaks have yielded valuable information about the environmental niche of *Histoplasma*, situations that may present risks for infection, and the importance of histoplasmosis as an occupational illness. A previous review found that approximately one-third of documented histoplasmosis outbreaks were work-related [11].

Examples of workers affected in previous histoplasmosis outbreaks include bridge workers, construction or demolition workers, farmers, landscapers or tree removal workers, and microbiology laboratory workers (Table 1) [11].

In general, environmental disruption of *Histoplasma* habitats is a key factor associated with histoplasmosis outbreaks. This disruption can be minor, such as simply walking on contaminated ground, or it can be relatively large, such as a construction site resulting in windborne dispersal of *Histoplasma* conidia infecting hundreds of people throughout a city. Some outbreaks also affected people infected at their workplace but not directly involved in the outbreak-initiating activities, for example, office workers infected after construction or renovation [11,43,57].

Disturbance of large accumulations of bird or bat droppings is a common feature, in approximately 40% of identified outbreaks [11]. Examples include outbreaks affecting workers scraping bird droppings from a bridge, shoveling bat droppings out of an attic, or cleaning chicken coops [28,32,44,46,58]. The mere presence of birds or bats, even without obvious accumulations of droppings, is noted in more than 75% of all histoplasmosis outbreaks and in 86% of work-related outbreaks, again indicating that even small environmental disruptions can pose a risk, and that the potential risk to workers extends beyond those directly involved with cleaning up droppings [11]. Other types of environmental disruption in histoplasmosis outbreaks include soil (i.e., digging or excavation, in one-third of outbreaks) or plant matter, such as cutting trees or wood, gardening, or landscaping [11]. Demolition, construction, or renovation precedes approximately one-quarter of histoplasmosis outbreaks [11].

These specific activities also appear to be potential exposure sources for non-outbreak-associated (i.e., sporadic) histoplasmosis cases, though to a lesser extent. However, occupational and recreational activities potentially associated with infection in sporadic cases are not typically assessed during routine public health surveillance. Enhanced surveillance interviews with histoplasmosis patients reported to public health authorities in nine states (Arkansas, Indiana, Kentucky, Louisiana, Michigan, Minnesota, Nebraska, Pennsylvania, and Wisconsin) in 2018–2019 revealed that 48% reported gardening, landscaping, or handling plants or trees, 37% reported digging soil, 28% reported participating in or being nearby construction, demolition, or renovation, and 24% reported handling bird or bat droppings [15]. Nearly a quarter of patients did not recall any of these exposures, and immunosuppressed patients reported fewer exposures than non-immunosuppressed patients [15]. Therefore, specific exposures seem to be less common or less obvious with sporadic cases than in outbreaks and determining whether such exposures are work-related can be difficult, particularly if people participate in recreational activities that also present a risk for *Histoplasma* exposure. Because of the increased risk for severe histoplasmosis manifestations among immunosuppressed people, people with certain underlying conditions should consider avoiding the types of activities described above in areas where *Histoplasma* is common.

Work-related histoplasmosis can occur in various settings. In general, frequent settings for histoplasmosis outbreaks have included buildings or outdoor structures, chicken coops or farms, and other outdoor areas, although reported chicken coop-associated outbreaks have not been consistently reported in published literature for nearly 70 years (Table 1) [11]. This could indicate a true reduction in these outbreaks over time related to implementation of prevention methods, or it could simply indicate the absence of a need to report these outbreaks because the risk has been so well-established. Regardless, farms and chicken coops likely continue to be common settings for *Histoplasma* exposure. This is supported by data indicating that histoplasmosis may be more common among residents of rural areas [15].

## 5. Preventing *Histoplasma* Exposures in the Workplace

**Figure 1:**
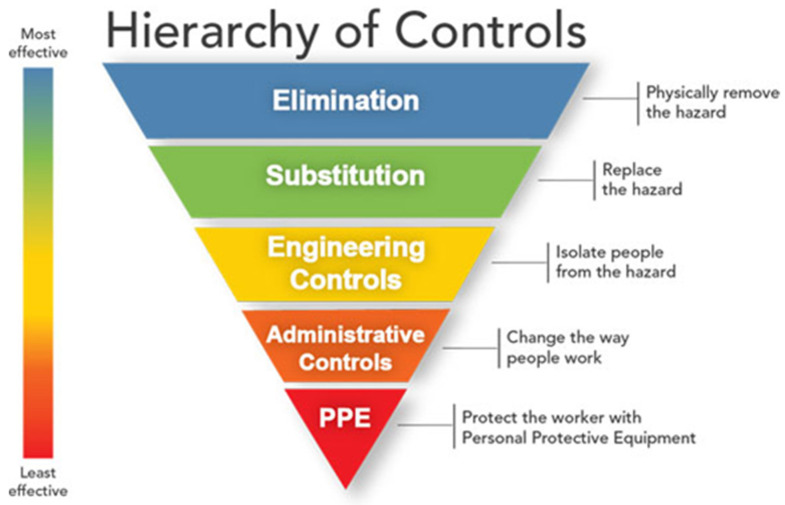
The hierarchy of controls for controlling exposures to occupational hazards. This framework is presented with the methods at the top of graphic as more effective and protective than those at the bottom. Source: National Institute for Occupational Safety and Health (NIOSH).

Occupational health and safety specialists use the hierarchy of controls (Figure 1) to determine how to implement feasible and effective control solutions to occupational hazards. This framework can be used to prevent exposure to histoplasmosis in the workplace (Table 2) [61,62]. Elimination (removing the hazard) and substitution (replacing the hazard) are the most effective ways to reduce occupational hazards but can be difficult to implement for infectious agents such as *Histoplasma*. In some cases, large amounts of bird or bat droppings should be cleaned up by a professional company that specializes in handling hazardous waste. Engineering controls are physical changes to work processes to remove the hazard or place a barrier between workers and hazards. Administrative controls are methods that change the way the work is performed. Finally, personal protective equipment (PPE) provides a physical barrier between the worker and the hazard. PPE is considered the least effective control measure because it requires a comprehensive program and a high level of worker involvement and commitment for proper use [61].

Developing a site safety plan with input from management, employee representatives, and health and safety professionals, is an important step in minimizing workplace exposures [63]. A comprehensive plan includes the identification of potential hazards and a description of the necessary measures to prevent, control, and reduce those hazards. Measures should include engineering and administrative controls and use of PPE.

### 5.1. Elimination/Engineering Controls

#### 5.1.1. Excluding Bats or Birds from a Building

Because work-related exposure to *Histoplasma* often occurs during disruption of bird or bat droppings [2], following the hierarchy of controls, the best way to prevent exposure to *Histoplasma* is to prevent the accumulation of bird or bat droppings in the first place. There are recommended protocols for excluding bats and birds from buildings [64,65,66]. Sealing all entry and exit points in the building is the first step. For bats, additional steps may include installing lights in daytime roosting areas and constructing bat houses near former roosts. Ultrasonic devices and chemical repellents are not effective for eliminating bats from a roosting area [67]. For birds, additional steps may include using visual deterrents and noises, periodically applying nontoxic chemical bird repellents, and installing a mechanical anti-roosting system consisting of angled and porcupine wires made of stainless steel.

#### 5.1.2. Controlling Dust Generation and Aerosolized Dust

Once a roosting site has been discovered in a building or other location, exclusion plans should be made, and the extent of contamination should be assessed. Removing accumulations of bat or bird droppings may not always be the next step. Simply leaving the material alone may be the best option if human activity around the location is unlikely. However, if the decision is made to remove accumulations, it is important to consider factors such as the amount and location of the accumulated material, the structural integrity or soundness of the building or structure, weather conditions, and whether people are near it.

During the removal of the material, work practices and dust control measures that eliminate or reduce dust generation will lower risk of infection. For example, carefully spraying dry, dusty material with water instead of shoveling or sweeping can reduce the amount of aerosolized material [51]. Adding a surfactant or wetting-agent to the water might further reduce the aerosolization. An alternative method is to use an industrial vacuum cleaner with a high-efficiency filter to collect potentially contaminated material. Truck-mounted or trailer-mounted vacuum systems are recommended for areas with large accumulations of bat or bird manure.

Even in the absence of large accumulations of bat and bird droppings, *Histoplasma* conidia can be aerosolized during cleaning, construction, excavation, or demolition. Once airborne, conidia can be carried easily by wind currents over long distances and distributed indoors through the air handling unit or natural ventilation, and these conidia could infect people outside of the work site [8].

Water sprays or other dust suppression techniques can reduce the amount of dust aerosolized during construction, excavation, or demolition in regions where *Histoplasma* is common [54,68]. During windy periods or other times when typical dust suppression techniques are ineffective, earthmoving activities should be interrupted. It is most protective if all earthmoving equipment, such as bulldozers, have enclosed cabs with air-conditioning and HEPA filtration to protect their operators. Other protective measures include covering all truck beds carrying dirt or debris from a work site and having all trucks pass through a wash station before leaving the site.

#### 5.1.3. Disposing of Waste

Any material removed from a work site that might be contaminated with *Histoplasma* should be disposed of properly and safely and should not be moved to another area where it could still be a health hazard. It is important to follow state and local requirements for the removal, transportation, and disposal of potentially contaminated material. If local or state landfill regulations define material contaminated with *Histoplasma* to be infectious waste, incineration or another disposal method may also be required.

#### 5.1.4. Disinfecting Potentially Contaminated Material

There are no Environmental Protection Agency-approved products registered specifically as soil disinfectants or as being effective against *Histoplasma*. In past histoplasmosis outbreak settings, formaldehyde was used to decontaminate material contaminated with *Histoplasma* [47,69,70]. However, this is not recommended because formaldehyde can cause a variety of health problems [71].

### 5.2. Administrative Controls

#### 5.2.1. Posting Health Risk Warnings

Signs warning people of the health risk should be posted in areas known or suspected to be contaminated with *Histoplasma*, like bird or bat roosts, attics, or entire buildings that contain accumulations of bat or bird droppings.

#### 5.2.2. Hazard Communication and Training

Before starting an activity that could disturb any material that might be contaminated by *Histoplasma*, it is essential that workers understand the potential risks and how to protect themselves. OSHA’s Hazard Communication Standard requires employers to inform and train workers on potential work hazards and associated safe practices, procedures, and protective measures [72]. Recommended components of a written hazard communication program about histoplasmosis include signs and symptoms, risk factors, treatment, and how to prevent exposures.

### 5.3. Personal Protective Equipment

To protect employees from breathing contaminated air when effective engineering controls are not feasible or while being instituted, federal regulations require the use of respirators [73]. Respirators are devices designed to provide clean breathable air to the wearer.

To be effective, respirators must be National Institute for Occupational Safety and Health (NIOSH)-approved and properly selected and used. Workers must also be fit tested for tight fitting respirators and undergo training [73].

Although research is limited on the effectiveness of respirators in protecting workers from breathing in dust containing *Histoplasma*, respirators are expected to offer some level of protection because the diameter of *Histoplasma* conidia ranges from 1 µm to 5 µm [13,74,75]. NIOSH-approved respirators will collect all types of workplace aerosols, including airborne infectious organisms with very high efficiency [74,76].

Respirators must be selected based on:

- the level of risk for histoplasmosis while performing the job;
- the required assigned protection factor [73]. The assigned protection factor is the level of workplace protection that each class of respirators is expected to provide to employees when the employer implements a continuing, effective respiratory protection program;
- the advantages and disadvantages of each respirator class that provide the required assigned protection factor.

For medium risk activities involving soil disruption (e.g., demolition, excavation, farming), working with live poultry, or other birds (except where large accumulations of droppings exist, which pose a higher risk), the use of half-facepiece respirators including both filtering facepiece respirators and elastomeric respirators can be considered.

For high risk activities involving work at remediating sites with documented *Histoplasma*, or disrupting large accumulations of bird or bat droppings, removing trees or other plant material at large bird roosting sites, the use of a powered air-purifying respirators (PAPR) equipped with any of the approved filters (HE, PAPR100-N, and PAPR100-P) or a full facepiece respirator with 100 series filters may be more appropriate.

If PAPRs and N100 full facepiece respirators are not available, the use of half-facepiece respirators can be considered. However, it should be noted that these respirators only have a protection factor of 10 so they only provide 20% of the protection afforded by PAPRs and full facepiece air purifying respirators. The employer will need to determine whether this is enough protection depending upon the environment in which the wearer will be.

Eye protection (either eyecup or cover-type safety goggles) is recommended. Additionally, disposable protective clothing and shoe or boot coverings should be worn whenever regular work clothing and shoes might be contaminated with dust containing *Histoplasma* conidia. This can reduce skin and mucous membrane exposure as well as eliminate the likelihood of transferring conidia to places away from a worksite, such as a car or home. When spore-contaminated material is likely to fall from overhead, workers should wear disposable protective clothing (i.e., coveralls) with hoods [77]. Workers should wear disposable shoe coverings with ridged soles made of slip-resistant material to reduce the likelihood of slipping on wet or dusty surfaces. Since protective clothing can be more insulating than regular work clothing, precautions may need to be taken to control heat stress. After completion of work, workers should remove all protective clothing and shoe coverings and seal them in heavy duty plastic bags for disposal [78].

## 6. Laboratory-Acquired Histoplasmosis

Laboratory acquired histoplasmosis is an essential consideration for clinical and research laboratorians that handle clinical samples or cultures containing viable *H. capsulatum*. In the United States, reports in the peer-reviewed literature describe laboratory-acquired histoplasmosis since the early 1950s [79]. Laboratorian exposures can occur to both filamentous and yeast phases of *H. capsulatum*. Case reports have noted several routes of occupational exposure for the laboratorian and include the inhalation of respirable infectious *H. capsulatum* aerosols during laboratory procedures, accidental inoculation, and transmission to skin and mucous membranes [79,80,81,82,83].

The hierarchy of controls can be used as a framework to prevent exposure to *H. capsulatum* in laboratory settings. Although elimination and substitution are not practical in a clinical and research laboratory, the risk of worker exposure to *H. capsulatum* can be reduced through laboratorian knowledge of the facility laboratory safety manual, laboratorian training, medical surveillance, as well as the containment of microbial exposures through aseptic microbiological practices [79,80,84]. Handling clinical and culture samples in a biosafety level (BSL)-3 laboratory when available and a laminar flow Biological Safety Cabinet (BSC) while wearing appropriate PPE can prevent *H. capsulatum* exposures [80,84]. Additional approaches used to prevent exposure in the laboratory can include shrink-wrapping or taping culture plates closed, not performing slide cultures, and testing with molecular and proteomic approaches early in the culture of *Histoplama*. Laboratory exposure may also be challenging to determine as histoplasmosis infections could be subclinical with variable incubation periods [79,80,84] and occur in nonendemic regions. Workplace accidents and inadvertent exposures resulting in symptomatic histoplasmosis should be reported following the facility’s policies and procedures and prompt consultation with an occupational physician.

## 7. Public Health Implications

Discovering risk factors for transmission and assessing hazards in the workplace could help employers plan disease prevention measures, such as implementing changes in work practices or an OSHA-compliant respiratory protection program. Including the systematic collection of occupational information as part of histoplasmosis surveillance might facilitate identifying future workplace-associated outbreaks. Capturing information on both industry and occupation for both sporadic and outbreak-associated histoplasmosis cases can further inform public health workers on those specific job risk factors needing further assessment. Unfortunately, industry and occupation information are not collected in all states where histoplasmosis is considered endemic, and states have disparate ways of collecting responses. Examples include “checkboxes” for industries or occupations of interest, “free-text fields” for occupation, and “pick-lists” of job categories based on standard classifications systems or customized lists. Although checkboxes and pick-lists may be efficient, they may be incomplete and miss capturing at-risk workers.

To improve data collection in surveillance systems, the NIOSH Surveillance Program at the Centers for Disease Control and Prevention (CDC) recommends that occupational questions should be standardized, information on both industry and occupation should be collected, and data should be analyzed with standard coding schemes to monitor disease trends in specific industries or occupations and protect workers’ health [85,86]. Other helpful information for histoplasmosis surveillance includes employer name, work location, job duties, and questions about specific types of exposures and protective measures taken.

In addition, employers should provide employee rosters to public health agencies to assist in identifying histoplasmosis cases when necessary. Project owners and employers should also report cases of histoplasmosis among their workers to public health agencies. Employers are currently required to report illnesses resulting in hospitalizations among workers to OSHA programs, and public health agencies should establish agreements with occupational safety and health agencies to share data for surveillance purposes. Outreach in both non-endemic and endemic areas can prompt healthcare providers to recognize potential work-associated histoplasmosis.

## 8. Conclusions

Histoplasmosis should be considered when workers in industries or occupations at increased risk have symptoms compatible with the disease. Communication and cooperation between clinicians and public health practitioners is important to identify work-related clusters of histoplasmosis. Consideration of occupational risk factors and controlling exposures to workers according to the hierarchy of controls will help prevent disease transmission in the workplace. Future research on the effectiveness of interventions to minimize worker exposures to *Histoplasma* is needed and should include environmental mitigation and respiratory protection.

## Figures and Tables

**Table 1:** Previously reported histoplasmosis outbreaks affecting workers.

Ref	Location	Month and Year	No. Cases	Activities and Setting	Type of Workers Affected
[26,27]	Plattsburg, New York	November 1938	23	Demolition and shoveling bird droppings from the roof of a school building	Works Progress Administration workers
[28]	Camp Crowder, Missouri	May 1943	40	Cleaning abandoned chicken coops, homes, and barns	Army members
[29]	Camp Gruber, Oklahoma	March 1944	27	Entering an abandoned storm cellar and chopping wood	Army members
[30]	Warrenton, North Carolina	May 1947	7	Church renovation	Carpenters and a contractor
[31]	Cincinnati, Ohio	July 1947	12	Exposure to bird droppings at an abandoned water tower	Not specified
[32]	Anne Arundel County or Calvert County, Maryland	August 1951	2	Scraping bird/bat droppings off bridges	Bridge workers
[26]	Mandan, North Dakota	February 1952	4	Demolishing a school building and removing bird droppings	Railroad workers
[33]	Johnstown, New York	November 1954	2	Cutting down a decayed tree	Lumberjacks
[34]	North Carolina	1956	2	House renovation	Not specified
[35]	Southwestern Minnesota	1956	5	Church renovation	Workers who cleaned and installed new window wells
[36]	Walworth, Wisconsin	August 1956	19	Excavation for water and sewer lines while constructing a new house	Construction workers
[37]	Lexington, Kentucky	October 1960	7	Removing bird droppings at a water tower	Not specified
[38]	Mason City, Iowa	August–September 1962	28	Clearing trees and bushes at a bird roosting site	Workers who cleared vegetation
[39]	Northwest Illinois	June 1967	12	House renovation	Construction workers
[40]	Jane Lew, West Virginia	March 1968	4	Building renovation and digging for gas lines	Maintenance crew, investigators, and laboratory workers
[41]	Jacksonville, Texas	March 1971	2	Bulldozing a blackbird roost	City workers
[42]	Aquas Buenas Caves, Puerto Rico	May 1973	4	Digging for fossils in a cave	Students and teachers
[43]	Hot Springs, Arkansas	July 1975	68	Clearing bird droppings from courthouse roof	Construction workers and office workers
[44]	Southern Maryland	November 1977	13	Scraping bat droppings from a bridge	Bridge workers and epidemiologists
[45]	Bossier Parish, Louisiana	September 1977	6	Clearing bamboo from a bird roosting site	Temporary laborers
[46]	Tennessee	September 1977	2	Cleaning bat droppings from a bridge	Bridge workers
[47]	Pittsfield, Illinois	April 1980	29	Disruption of bat droppings during renovation of a school building	Heating/ventilation worker and school employees
[48]	Rogers City, Michigan	January 1980	138	Exposure to a pulley stored in a bird nesting area	Limestone quarry workers
[49]	Rockville, Maryland	January 1987	13	Renovation of a bat-infested house	Construction workers
[50]	Lares, Puerto Rico	September 1987	4	Uprooting marijuana plants	Police officers
[51]	Muskegon County, Michigan	October 1993	44	Sweeping bird droppings from roof at a pulp paper factory	Factory workers
[52]	30 miles west of Richmond, Virginia	June 1994	72	Moving a pile of dirt and debris	Prison employees and inmates
[53]	Eastern Kentucky	June 1995	19	Disruption of bat guano during demolition of abandoned building	Demolition workers
[54]	Macon County, Illinois	May 2001	6	Moving soil and clearing trees at a landfill	Landfill workers
[54]	Iroquois County, Illinois	August 2003	5	Bridge repair	Bridge workers
[55]	Blair, Nebraska	January 2004	108	Removal of contaminated soil excavated during a previous histoplasmosis outbreak	Agricultural processing plant workers
[56]	Des Moines, Iowa	November 2007	55	Construction and renovation at a state facility building	Construction workers and office workers
[57]	Iowa	October 2008	23	Demolishing a bat-infested attic	Construction workers
[58]	McLean County, Illinois	August–September 2011	8	Disrupting bat droppings during building restoration	Temporary laborers
[59]	Douglas County, Nebraska	June 2012	36	Cleaning bat droppings from a campsite	Camp counselors
[60]	Danville, Illinois	August 2013	85	Removal of trees where birds roosted	Prison employees and inmates

**Table 2:** Measures to prevent *Histoplasma* exposures in the workplace.

Hierarchy of Controls	Prevention Measure
Elimination	Excluding bats or birds from a building
Engineering controls	Controlling dust generation and aerosolized dust Disposing of waste
Administrative controls	Developing site safety plan Posting health risk warnings Hazard communication and training
Personal protective equipment (PPE)	NIOSH-approved respirators Other PPE: eye protection, gloves, protective clothing, shoe/boot coverings
